# Insights on the Pathogenesis of *Mycobacterium abscessus* Infection in Patients with Cystic Fibrosis

**DOI:** 10.3390/jcm14103492

**Published:** 2025-05-16

**Authors:** Mai Basher, Michal Gur, Michal Meir

**Affiliations:** 1 Rappaport Faculty of Medicine, Technion–Israel Institute of Technology, Haifa 3525433, Israel; maibasher11@gmail.com (M.B.); m_gur@rambam.health.gov.il (M.G.); 2 Clinical Research Institute Rambam (CRIR), Rambam Health Care Campus, Haifa 3109601, Israel; 3 Pediatric Pulmonary Institute and CF Center, Rappaport Children’s Hospital, Rambam Health Care Campus, Haifa 3109601, Israel; 4 Pediatric Infectious Diseases Unit, Rappaport Children’s Hospital, Rambam Health Care Campus, Haifa 3109601, Israel

**Keywords:** cystic fibrosis (CF), *Mycobacterium abscesus* (Mab), non-tuberculous mycobacteria (NTM), host–pathogen interaction, virulence

## Abstract

People with CF (pwCF) have a significant risk for pulmonary infections with non-tuberculous mycobacteria (NTM), particularly *Mycobacterium abscessus* (Mab). Mab is an emerging pathogen, which causes pulmonary infections in patients with chronic lung diseases, particularly CF; Mab pulmonary disease leads to progressive pulmonary dysfunction and increased morbidity and mortality. Despite advances in CF care, including CFTR modulators (CFTRm), Mab continues to pose a therapeutic challenge, with significant long-term medical burden. This review provides insights into the complex host–pathogen interplay of Mab infections in pwCF. It provides a detailed overview of Mab bacterial virulence factors, including biofilm formation, secretion systems, the virulence-associated rough morphotype, and antibiotic resistance mechanisms. This review also summarizes features conferring susceptibility of the CF host to Mab infections, alongside the contribution of the CF-host environment to the pathogenesis of Mab infection, such as antibiotic-derived microbial selection, within-host mycobacterial evolution, and interactions with co-pathogens such as *Pseudomonas aeruginosa* (PA). Finally, the therapeutic implications and novel treatments for Mab are discussed, considering the complex host–pathogen interplay.

## 1. Introduction

*Mycobacterium abscessus* (Mab), an environmentally ubiquitous non-tuberculous mycobacteria (NTM), is an emerging pathogen known to cause severe soft tissue infections, health care-associated blood stream infections, and devastating lung disease [1,2]. Mab infections are typically chronic and locally progressive [3], yet may disseminate, mostly in patients with immune-deficiency [4]. Mab pulmonary disease is uncommon among otherwise healthy and immune-competent individuals, yet is becoming increasingly prevalent in patients with chronic lung disease, especially people with cystic fibrosis (pwCF) [5].

Cystic fibrosis (CF) is an autosomal recessive disorder caused by pathogenic mutations in the CF transmembrane conductance regulator (CFTR) gene, encoding a chloride channel responsible for anion transport across epithelial cells [6,7]. CFTR dysfunction affects multiple organ systems lined with epithelial cells, including the airways, the gastrointestinal tract and the reproductive organs [8,9]. In the respiratory epithelium, CFTR mutations result in abnormally viscous luminal secretions, leading to a reduced ability to clear mucus [10]. The accumulation of excessive mucus plugs and decreased mucociliary clearance lead to inflammation and infection, which are the hallmarks of the disease [10,11]. 

PwCF are at a significantly higher (1000-fold) risk for NTM respiratory infections [12], especially Mab infections, with an estimated annual prevalence of 4.8% [13]. Mab infections in pwCF are considered most devastating, leading to progressive pulmonary deterioration [14], significant morbidity and increased mortality [12,15]. Pulmonary infections often persist for years, requiring long-term therapy with multiple antibiotics [1]. Despite advances in CF therapy, including CFTR modulators (CFTRm), Mab infections continue to lead to great morbidity with long-standing therapeutic burden among pwCF [12,15].

PwCF present a setting of altered physiology, disrupted anatomy (e.g., bronchiectases), impaired mucociliary clearance, dysregulated immune responses, and chronic inflammation—all of which contribute to the increased susceptibility to respiratory pathogens, including Mab. Pathogen virulence factors exploit these host abnormalities to establish persistent infections and drive inflammation. A well-recognized example is the capacity of Mab to form biofilms, which promote microbial persistence and resistance to both immune clearance and antibiotics [16]. This feature is shared by key CF pathogens such as *Staphylococcus aureus* [17] and *Pseudomonas aeruginosa* [18]. In addition, Mab possesses unique mycobacterial virulence traits, including specialized type VII secretion systems (T7SSs), that facilitate immune evasion [19], and a rough colony morphology associated with heightened inflammatory responses and increased pathogenicity [20]. Compounding the complexity, pwCF frequently harbor polymicrobial infections, with interspecies interactions further shaping the airway environment and influencing host–pathogen dynamics [21,22,23]. This review explores the multi-faceted interplay between Mab and the CF host, highlighting implications for the current and emerging therapeutic strategies. A deeper understanding of these interactions is critical for the development of effective interventions targeting this formidable pathogen in the CF population.

## 2. The Immune Response to Mab Infection

Mab is able to infect various cell types, including epithelial cells, macrophages, and neutrophils [24]. In the respiratory system, Mab infects the host mainly through the airways, where it comes in direct contact with the mucosal surface and the innate immune components. Mab initially creates a biofilm on the surface of the lung epithelial mucosa, then invades the lung epithelial cells, in which it proliferates [25,26]. Within cells, Mab alters the cellular metabolism, changing the membrane permeability, compromising mucosal integrity, and allowing the translocation of bacteria into the sub-mucosal space [27].

Within the respiratory tract, Mab activates the innate immune system, leading to recruitment of dendritic cells, neutrophils, and macrophages [28,29,30,31]. Initially, Mab activates the macrophage through signaling cascades related to the dual recognition of Toll-like receptor (TLR) and Dectin 1, a pattern recognition C-type lectin receptor [32]. Macrophage internalization induces the secretion of pro-inflammatory cytokines and enhanced phagocytosis [32,33,34]. Following phagocytosis, the bacteria-containing vacuole fuses with the lysosome, leading to bacterial degradation and killing.

A key element of the immune response to mycobacterial infection is the formation of the granuloma, an organized aggregation of immune cells (including lymphocytes, macrophages, and epithelioid cells), that aids in the local maintenance and control of tissue infection [35]. Animal model studies of Mab infections support a significant role of CD4+ lymphocytes and cytokines, including interferon-γ (IFN-γ) and tumor necrosis factor (TNF), in stabilizing the Mab-containing granuloma [35,36,37].

Nitric oxide (NO) also plays an important role in the innate immune response and in the host defense against mycobacterial infection. In response to interferon type-I signaling, Mab-infected macrophages produce NO, which restricts intracellular replication of the bacteria. Animal-model studies have shown that disruption of this pathway leads to increased Mab proliferation and severe infections [37,38].

Human diseases, namely primary or secondary immune deficiency, have led to insights into the role of additional elements of the immune system in controlling mycobacterial infection. Mendelian susceptibility to mycobacterial disease (MSMD) relates to a group of inherited errors of immunity, conferring selective susceptibility to infections by otherwise low-virulence mycobacteria. These single-gene dysfunctions mostly affect NFkB, IFN-γ, and interleukin (IL)-12 or IL-23 signaling, suggesting their pivotal role in controlling mycobacterial infections [39]. Treatment with anti-TNFα biological agents is also associated with increased susceptibility to NTM, supporting the role of TNFα in controlling these infections [40].

In specific hosts, Mab is able to overcome the complex immune mechanisms owing to multiple virulence factors enabling its survival and proliferation. Section 3 will discuss the virulence factors characterizing Mab and contributing to its ability to cause progressive disease.

## 3. Mab Virulence Factors

Mab is known to harbor multiple and complex virulence factors, allowing its survival within host cells, evasion of the host-immune system, and development of antibiotic resistance and tolerance [1,19].

### 3.1. Type VII Secretions Systems

Secretion systems are protein complexes located on bacterial cell membranes that enable the transport of proteins across the cellular membranes. In mycobacteria, type VII secretion systems (T7SSs) have five subtypes (ESX-1 to ESX-5), and are considered a key factor in the host–pathogen interactions [19]. In Mab, ESX-3 and ESX-4 play a crucial role in promoting mycobacterial pathogenicity [41,42]. They both promote the ability of Mab to survive within macrophages, preventing fusion of the phagosome with acidic compartments and avoiding phagosome maturation. They also likely have a substantial role in facilitating Mab escape into the cytosol, aiding in bacterial multiplication and dissemination [42,43].

### 3.2. Mab Rough (R) Morphotype

One of the distinct features associated with Mab virulence is related to the transition of colony morphology from a smooth (S) to rough (R) morphotype. This transition is primarily driven by a reduction in the levels of glycopeptidolipids (GPLs) in the outer membrane, and is related to several governing genes and transcription factors [44]. The R morphotype is known to be more virulent in both pre-clinical models and in human disease, and is associated with severe infections, increased inflammatory response, and poor clinical outcomes [20,29,44]. In pwCF, the Mab may exhibit the R or S morphotype, or in some cases, a mixture of both [45]. 

Several characteristics of the R colony morphotype likely contribute to its heightened virulence. Firstly, R isolates effectively evade phagocytosis and subsequent phagocytosis-induced microbial killing. In host tissues, these isolates proliferate in aggregates that form extracellular structures known as serpentine cords, which are too large to be efficiently internalized by phagocytes [44,46,47]. When phagocytosed, R isolates can persist intracellularly by triggering a type I interferon (IFN-I)-mediated escape into the cytosol [48], reducing their susceptibility to reactive oxygen species [49], and promoting macrophage apoptosis [28,50]. Moreover, R isolates are capable of replicating within macrophages and amplifying the host inflammatory response [51,52,53], thereby exacerbating the inflammation-associated pathology and contributing to tissue damage and dysfunction.

### 3.3. Biofilm Formation

Biofilm describes an aggregate of bacterial cells, that firmly attach to a surface or human tissue and enclosed in an extracellular matrix, mostly polysaccharides [54]. Biofilms exhibit highly viscoelastic properties; therefore, they resist clearance from the lungs and contributing to the persistence of chronic pulmonary infections. Overall, the ability of Mab to create biofilms is a major feature driving the pathogenesis of Mab pulmonary infection in patients with chronic lung diseases [25,26].

Mab biofilms have been linked to pathogen adaptation within the host and increased virulence. Bacteria within biofilms are less impacted by antibiotics, owing to limited physical contact with harmful agents within their environment, and to altered bacterial metabolism and growth rate [54]. These changes within Mab biofilms lead to microbial tolerance [55,56], accounting at least in part for the high antibiotic failure rates in Mab antimicrobial therapy, and the discrepancy between susceptibility testing in vitro and clinical response [57].

## 4. The CF Host

As mentioned above, the pathophysiology of CF is governed by the dysfunction, or lack of function, of the CFTR protein. In the respiratory system, CFTR dysfunction leads to the production of thick, viscous mucous, leading to mechanical obstruction, increased inflammation, and reduced ability to clear respiratory pathogens.

Continuous and chronic infection and inflammation damage the airway architecture, leading to the formation of bronchiectasis. While bronchiectasis is not specific to CF, it is a common pathology in pwCF, further contributing to the impaired clearance of secretions and increased risk of infections [58,59,60,61]. Bronchiectasis and NTM infections are strongly correlated; the presence of bronchiectasis is a risk factor for NTM infection, while NTM infection leads to the worsening of bronchiectasis within the lungs [62].

Susceptibility to infection in pwCF may also result from localized metabolic imbalances, particularly alterations in the lipid composition of airway epithelial cells [63]. Recent studies have identified significantly reduced sphingosine levels in bronchial and alveolar epithelial cells, as well as in macrophages, from both CF mouse models and human patients. This sphingosine deficiency has been implicated in the increased vulnerability of CF tissues to Mab infection. Furthermore, exogenous sphingosine treatment has been shown to enhance bacterial clearance, underscoring its potential as a therapeutic agent [64].

### Hyperinflammation and Immune Dysregulation

In the CF airways, a hyper-inflammatory state occurs due to excessive immune responses, regardless of (and in addition to) infection [65,66,67,68]. CF airways are noted for increased neutrophil recruitment and the secretion of pro-inflammatory cytokines, such as TNFα, IL1β, IL-6, 8, 17, 33, and granulocyte–macrophage colony-stimulating factor (GM-CSF). The release of inflammatory mediators results in excessive and on-going inflammatory responses, neutrophilic inflammation, and tissue damage [69,70,71]. Furthermore, the repair of damaged airway cells of pwCF is notably slower compared to non-CF airway cells [65].

CFTR is expressed not only in epithelial cells, but also in immune cells such as monocytes and neutrophils. Several studies have suggested that CFTR dysfunction contributes to immune dysregulation, particularly impairing the ability of phagocytes to control and clear intracellular bacterial infections. CFTR-deficient phagocytes exhibit impaired reactive oxygen species (ROS) generation, compromising their bactericidal function and facilitating intracellular bacterial persistence [72]. Similarly, CFTR dysfunction in neutrophils has been associated with altered immune responses. In vitro studies have shown that CFTR-deficient neutrophils display enhanced and prolonged inflammatory activity, contributing to chronic neutrophilic inflammation [73,74]. Given the emerging evidence that neutrophils play a critical role in host defenses against Mab [28,75], these immune impairments may contribute to the increased susceptibility of pwCF to Mab infection [28,30,72].

The extent to which CFTR dysfunction directly contributes to the heightened susceptibility to infections in pwCF remains a topic of active investigation and debate [76,77]. While, as mentioned above, intrinsic defects in CFTR function have been implicated in impaired immune responses, particularly within macrophages, several CFTR-independent factors associated with the altered airway environment in CF may also compromise macrophage function. These include biophysical abnormalities such as mucin hyper-concentration and airway obstruction [78,79]. In addition, sustained exposure to neutrophil-derived inhibitory mediators has been shown to contribute to macrophage exhaustion and dysfunction [80], further impairing the host’s ability to clear pathogens effectively.

The development of CFTRm therapy may provide valuable insights into the ongoing debate regarding intrinsic versus environmental contributions to immune dysfunction in CF. While CFTRm therapy has been well documented as improving the pulmonary function, nutritional status, and quality of life, its impact on inflammation remains incompletely understood [81]. The effect of CFTRm on macrophage function has shown conflicting results—either showing modest improvement [82] or non-differential improvement in CF and non-CF patients [83]. Despite these modest or inconsistent effects on inflammation, a recent multi-center study demonstrated that treatment with elexacaftor/tezacaftor/ivacaftor (ETI) led to the successful eradication of NTM in nine out of fifteen pwCF, seven of whom were infected with Mab. Several mechanisms have been proposed to explain the impact of CFTRm on Mab. Firstly, ETI enhances mucociliary clearance in pwCF, promoting the removal of bacteria—including NTM—from the airways [84]. Additionally, as previously discussed, Mab forms biofilms that contribute to localized oxygen depletion. In vitro studies have shown that Mab within biofilms becomes non-replicative and exhibits increased resistance to antibiotics such as amikacin, imipenem, cefoxitin, and moxifloxacin. Therefore, the improved hydration of the CF mucus induced by ETI may facilitate greater antibiotic penetration and, consequently, more effective eradication of Mab [55].

Larger studies are needed to define the effect of CFTRm on Mab infection in pwCF eligible for this therapy.

## 5. The Host–Pathogen Interaction

### 5.1. Within-Host Pathogenic Adaptation

Mab has transitioned from an environmentally ubiquitous, non-pathogenic organism to a clinically significant opportunistic pathogen. Genomic analyses of clinical isolates, particularly from pwCF, indicate ongoing within-host evolution. In a study by Bryant et al. [85], recurrent mutations were identified in specific genetic loci across multiple patients, suggesting the convergent evolution of adaptive traits. These loci include genes involved in the S-to-R morphotype transition, macrolide resistance, cell wall biosynthesis, and global regulatory pathways. The accumulation of such mutations is associated with phenotypic changes that enhance bacterial persistence, promote survival in the hostile host environment, and confer increased tolerance to antibiotic and metabolic stress [35,85,86].

In addition to genetic mutations, several studies have proposed that Mab undergoes epigenetic adaptations in response to the unique conditions of the CF airway [87]. Transcriptomic and proteomic analyses suggest that metabolic reprogramming occurs in response to altered nutrient availability, microbial competition within the CF lung microbiome, and sustained antibiotic exposure. These metabolic adaptations include enhanced biofilm formation and cell wall remodeling, which in turn lead to altered antibiotic susceptibility and increased tolerance—facilitating Mab persistence within the CF airway niche [88,89].

The chronic inflammatory environment, host immune responses, and repeated antibiotic treatments characteristic of the CF lung likely exert a selective pressure that shapes both the genomic evolution and epigenetic reprogramming of Mab, thereby enhancing its pathogenic potential. As CFTRm modify the airway environment in CF, it is of considerable scientific interest to investigate how these therapies influence the genetic and epigenetic adaptation of Mab.

### 5.2. Co-Pathogens in CF Airways

The CF airways are inhabited by a variety of microbes, including bacteria, fungi, and viruses, that may lead to infection, inflammation, or both [90]. The interaction between pathogens within the microbiome of the CF-host likely play an important role in shaping the pathological outcomes of infection.

*Pseudomonas aeruginosa* (PA) is one of the most prevalent pathogens causing respiratory infections in pwCF. It is characterized by its wide range of virulence factors, and high resistance to antibiotics, making it challenging to eliminate from infected individuals, especially in pathologic airways, such as in pwCF [91]. It has been shown that 58–78% of the patients infected with NTM are co-infected with PA [14]; this is possibly related to antibiotic selection, as the antibiotics targeting PA have been found to create competitive advantage promoting Mab survival [92].

The co-infection of PA and Mab complicates the clinical presentation and increases lung damage and inflammation, leading to worsening clinical outcomes. Nevertheless, the interaction between PA and NTM is not trivial, nor is it fully understood. PA is able to inhibit Mab growth within biofilms, and to affect protein expression and cell structure of Mab through secretion of quorum-sensing molecule biofilms [93,94]. In turn, Mab degrades and reduces the production of quorum sensing molecules by PA, potentially limiting its virulence [95,96]. Overall, the wholesome effect of Mab and PA co-infection within the CF host remains unclear and necessitates further investigation.

**Figure 1:**
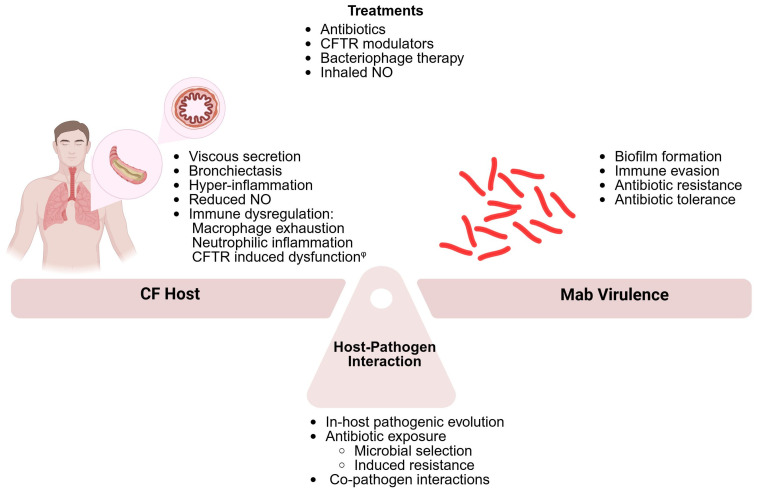
The interplay between the CF host and *Myobacterium abscessus.* Susceptibility to *M. abscessus* (Mab) infections among patients with CF (pwCF) is governed by host factors related to CF pathophysiology, by Mab virulence factors, and by their interaction. The exposure of Mab to the CF-airway environment, (including the host’s immune system, co-pathogens, and antibiotics) drive adaptation and Mab pathogenic evolution, further contributing to the interplay. Therapy for Mab infections involves prolonged courses of multiple antibiotics to prevent resistance and overcome tolerance. Novel therapies, such as CFTR modulators, bacteriophage therapy, and inhaled nitric oxide, may assist in overcoming infection. *^φ^* The role of CFTR in macrophage and neutrophil dysfunction is controversial and is of continued scientific debate.

Figure 1 describes the interplay between the CF host and Mab. As discussed above, this interplay is complex, and Mab infection in pwCF poses a therapeutic challenge. Section 6 will discuss the therapeutic implications, describing antibiotics and novel therapies aiming to control Mab pulmonary infection.

## 6. Antibiotic Treatment in Mab Infection

Mab may transiently, intermittently, or permanently reside within the lungs of pwCF, representing asymptomatic infection. However, Mab can also cause progressive inflammatory lung damage, termed ‘Mab pulmonary disease’ (Mab-PD). It is crucial to distinguish between asymptomatic colonization and active infection. Current guidelines suggest antibiotic therapy for Mab pulmonary disease (PD), defined according to microbiological, clinical, and radiological criteria. These include pulmonary or systemic symptoms (considering the contribution of other CF pathogens), typical radiologic findings, and the isolation of Mab (in two sputum cultures, one sputum culture and one bronchial wash, or lung biopsy) [97].

The treatment of Mab PD is challenging, necessitating prolonged multi-drug regimens in order to prevent emerging resistance and recurring infection [97,98].

There are several well-established antimicrobial agents commonly used in the treatment of Mab PD [98]. The treatment regimen is divided into two main phases: an initial intensive phase and a subsequent continuation phase. During the intensive phase, oral macrolides, such as azithromycin, are combined with intravenous amikacin, along with additional intravenous antibiotics (such as tigecycline, imipenem, or cefoxitin). In the continuation phase, treatment involves oral macrolides and inhaled amikacin, supplemented by two to three additional antibiotics (including minocycline, clofazimine, moxifloxacin, or linezolid) [97,98].

The response to therapy should be assessed by clinical, radiographic, and microbiologic data. Sputum cultures should be obtained every 1–2 months, to document when cultures become negative [97], and treatment should continue for 12 months after culture conversion [1]. Drug toxicity should be assessed by regular visual and hearing examinations, as well as the monitoring of renal and liver functions [98].

The phenomenon of antimicrobial resistance (AMR) is a critical global health challenge, especially relevant in CF. PwCF receive the highest cumulative antimicrobial drug load in their lifetime; thus, the worldwide increasing proportions of antibiotic-resistant strains is particularly evident [99]. The creation of biofilms, impeding the effectiveness of common antimicrobials in reaching their intended targets, further augments the challenge of AMR in PwCF [100]. Besides the triad *P. aeruginosa*, *Staphylococcus aureus*, and *Haemophilus influenza*, pathogens like *Achromobacter*, *Burkholderia complex*, and NTM display intrinsic resistances to currently available antibiotics, rendering the therapy of lung disease even more difficult [99].

Mab exhibits an array of antimicrobial resistance mechanisms against initially active antimicrobials. The ability to develop these resistance mechanisms, especially in pwCF, renders treatment additionally challenging and calls for alternative therapeutic approaches and novel therapies. Table 1 presents the mechanism of action and resistance mechanisms of antibiotics used to treat Mab PD. Notably, the antibiotics are not specific to Mab; however, as mentioned earlier, treatment includes multi-drug combinations for prolonged periods.

## 7. Novel Therapies

The prolonged and complex antibiotic regimens used to treat Mab, along with the high failure rates [57] raise the need to explore alternative therapies. Section 7.1 will discuss the non-antibiotic therapies that have emerged in recent years to treat Mab.

### 7.1. Nitric Oxide (NO)

CF airways are characterized by low NO. Suggested mechanisms are the thick mucus layer that prevents NO from reaching the bronchial lumen; another hypothesis is the shortage of substrates like L-arginine, essential for NO production. Decreased NO production may contribute to altered defense mechanisms and, thus, promote lung damage [113].

NO exhibits broad reactivity and rapid diffusion properties through lipid membranes and biological liquids; it has a short half-life (seconds) in a physiological milieu. Originally approved by the Food and Drug Association (FDA) to treat neonatal respiratory distress [114]; subsequent in vitro studies have shown that NO possesses antimicrobial activity against a wide variety bacteria, viruses, fungi, helminthes, and parasites [99].

In a phase 1 study conducted in eight adult CF patients, inhaled NO was tolerated well and decreased the bacterial burden in CF airways (not specific to Mab). Intermittent inhalations prevented the formation of toxic NO_2_ levels and methemoglobinemia. Mild side effects, such as dry mouth, were attributed to the drug; however, no serious adverse events necessitated premature study termination [99]. Subsequent small clinical studies showed initial positive results in pwCF and Mab infection. Patients tolerated the therapy well, with a reduced bacterial load of Mab, improved FEV1 and 6-min walking distance, and no serious adverse events [115,116]. It should be noted that this therapy is not widely available, and patients should be closely monitored. In these studies, inhaled NO was given as add-on to anti-Mab therapy and administered in a hospital setting under medical supervision. Further, larger studies will help understanding the feasibility of this treatment, and its ability to shorten or simplify current antibiotic regimens.

### 7.2. Bacteriophage Therapy

Bacteriophages are viruses that infect bacteria, mostly in a strain-specific manner or affinity. They are the most abundant microorganisms, distributed in soil, water, and air in different ecosystems and surfaces, both inside and outside human and animal bodies [113]. They attack the bacterial wall, reproduce their genome, and cause lysis of the host cell. First identified in the 1900s, the bacteriophages have gained interest in recent years, with the emergence of multi-drug resistance (MDR) bacteria; however, their use has been limited by inappropriate phage selection and preparation [117].

Therapeutic bacteriophages are pathogen-specific and are safe for human tissues. Bacteriophage therapy has to be tailored individually for each patient, involving the selection and preparation of specific phage species that are effective against the targeted Mab strain [117]. In a recent clinical study, mycobacterium isolates from 200 culture-positive patients with symptomatic disease were screened for phage susceptibilities. Twenty patients received phages administered intravenously, by inhalation, or both, for six months. All patients continued their previous antimycobacterial treatment with at least two drugs. Favorable clinical or microbiological responses were observed in 11 patients, while 8 patients developed neutralizing antibodies after initiation of therapy; no significant adverse events were observed [118].

Gorzynsky et al. investigated the phage–mycobacteria interaction and the synergism of phage–antibiotic combinations. They discovered the major phage receptor in mycobacteria; and found that phage lytic efficiency is altered by environmental conditions. Interestingly, they developed a set of phages that altered the multi-drug efflux pump function in Mab; the combination of these phages with antibiotics significantly decreased the number of viable bacteria, compared to antibiotics or phages alone [119].

In summary, bacteriophage therapy may be adventitious for pwCF with Mab infection; while initial results are encouraging, further studies are warranted to assess the feasibility of its widespread use. Currently, an open-label multi-center study (the POSTSTAMP study) is ongoing, treating 10 pwCF with treatment-refractory Mab-PD [119].

### 7.3. Other Therapies

#### 7.3.1. Host-Directed Therapies (HDTs)

HDTs are small molecules, specifically targeting the host immune responses to either enhance host immunity, modulate inflammation to reduce lung tissue destruction, and kill or contain Mab [120]. Resveratrol is an agonist of Sirtuin 3, known to play a critical role in host defenses against Mab; in mice and zebra fish infected with Mab, resveratrol significantly decreased Mab growth and attenuated inflammation and tissue damage [121]. Intravenous administration of mesenchymal stem cells in lungs and spleen of Mab-infected mice promoted antibacterial responses in macrophages via NO production [122]. A major advantage of HDTs is that they can be introduced as adjunct therapies in combination with classical antibiotics, maximizing the effect and shortening the length of therapy [120].

#### 7.3.2. Drug Repurposing

In the last decade, drug repurposing has emerged as an attractive strategy. For example, some antimalarial drugs, such as mefloquine which inhibits mycolic acid biosynthesis, have shown significant activity against Mab. Drugs such as disulfiram, used to treat alcohol dependence, or clomiphene citrate, utilized to treat women’s infertility, have demonstrated potent antimycobacterial activity. Despite their potential, the use of repurposed drugs against Mab faces challenges due to multiple resistance or tolerance mechanisms [120].

## 8. Omics in NTM Lung Disease in CF

Recent advances in omics technology has enabled the characterization of the microbial and metabolic features of the CF host. Proteomic and transcriptomic analyses have identified specific protein and mRNA biomarkers that correlate with impending pulmonary exacerbations in pwCF [123]. Additionally, the genomic profiling of the CF airway microbiome has been shown to correlate with therapeutic response [23]. Integrating meta-genomic and meta-transcriptomic data with microbial counts may allow an accurate assessment of species abundance and activity, informing targeted antibiotic therapies [120]. Altogether, these studies suggest a benefit to tailored approach of personalized therapy but also identify possible targets for intervention in attempt to diminish the infection- and inflammation-promoting environment.

Omics technologies have also been employed to facilitate the prediction and diagnosis of NTM infection in pwCF. Biomarkers identified in airway secretions and peripheral blood have demonstrated significant potential for both diagnostic applications and monitoring of therapeutic responses [124]. Phylogenomic analyses have been found to help trace the evolution and transmission of Mab strains within CF populations [125]. Furthermore, integrative analyses combining metabolomic and microbiome data have successfully distinguished pwCF with NTM infection from those without. However, it remains unclear whether the metabolic changes are secondary to NTM infection, or rather present a metabolic environment promoting NTM disease [126].

## 9. Conclusions and Future Directions

Pulmonary infections caused by Mab in pwCF involve complex host–pathogen interactions. These interactions encompass the inherent vulnerabilities of the CF host, the virulence factors of Mab, and its ability to adapt to the unique environment of the CF airway. A comprehensive understanding of these dynamics is critical in addressing Mab pulmonary infections in pwCF. This need is underscored by the high failure rates associated with antibiotic regimens, which primarily target bacterial inhibition. Moreover, antibiotic failure often incurs additional costs, including significant adverse effects and the promotion of antimicrobial resistance.

Although phage therapy has demonstrated enhanced antibacterial efficacy against persistent Mab infections, its clinical application is currently limited by accessibility and logistical constraints. Alternatively, therapies that target the host–pathogen interaction by modulating host factors have shown more encouraging outcomes. Among these, CFTRm are particularly notable. In addition to improving respiratory function and nutritional status, CFTRm have shown potential in facilitating the clearance of mycobacteria from the sputum. However, it remains to be determined whether CFTRm will achieve the sustained eradication of Mab from the airways of pwCF or inadvertently contribute to the further evolution of pathogenic traits.

Immune dysregulation and hyper-inflammatory state in pwCF has been long recognized. However, there is a continued scientific question examining the role of CFTR-induced immune dysfunction versus immune dysregulation secondary to chronic infection and perpetual inflammation. In vitro CF models, such as organ-on-chip models, have been able to demonstrate an epithelial cell response and immune-cell response in an airway-simulating structure [127,128]. Such models may be valuable tools to delineate the role of CFTR in immune dysfunction.

Preliminary investigations into emerging therapies that modulate the host immune response, along with omics-based approaches that leverage airway environmental changes for the diagnosis and treatment of Mab pulmonary infections, underscore the practical diagnostic and therapeutic potential of understanding the host–pathogen balance.

The outstanding research questions (see Table 2) include the anticipated impact of CFTRm therapy on Mab pathogenicity and the precise contribution of immune dysregulation to increased susceptibility to infection. Such studies and others lay the groundwork for further development of both pathogen- and host-aimed novel therapies, required to overcome this significant infection.

## Figures and Tables

**Table 1:** Antibiotic resistance mechanisms in Mab.

Antibiotics Active Against Mab	Antibiotic Class	Mechanisms of Action	Resistance Mechanisms
Azithromycin Clarithromycin	Macrolides	Inhibit bacterial protein synthesis by binding to the 50S subunit of the bacterial ribosome	Synthesis of erythromycin resistance methylase 41 (erm 41)—modifies the ribosomal binding site through methylation of the 23S rRNA [101]
Amikacin	Aminoglycosides	Inhibits bacterial protein synthesis by binding to the 16S rRNA of the 30S ribosomal subunit [98,102,103]	Aminoglycoside-modifying enzymes, such as Eis2 and AAC(2′)-II—prevent binding to the ribosomal unit [104]
Cefoxitin	Cephalosporins	β-lactam antibiotics—inhibit cell wall synthesis	Decreased cell wall permeability; production of β-lactamase enzymes [105]
Imipenem Meropenem	Carbapenems		
Tigecycline Minocycline	Tetracyclines	Inhibit bacterial protein synthesis, by binding to the 30S ribosomal subunit [106]	Tetracycline-modifying enzymes, e.g., monooxygenases—inactivate the antibiotic [107]; efflux pumps—actively expel antibiotics from the bacterial cell [108]
Moxifloxacin	Fluoroquinolones	Inhibits bacterial DNA replication by binding to the DNA gyrase and topoisomerase IV enzyme complexes [109]	Mutations in the antibiotic target enzymes—reduce binding affinity, limiting antibiotic access to the binding site [110]
Linezolid	Oxazolidinones	Inhibit bacterial protein synthesis by binding to the P site of the 50S ribosomal subunit.	Alterations in the 23S rRNA—reduce binding affinity [111]
TMP-SMX	Sulfonamides	Bind the enzyme dihydropteroate synthase enzyme therefore preventing synthesis of folic acid	Modifications in the dihydropteroate synthase enzyme—reduce binding affinity [112]

TMP-SMX—trimethoprim-sulfamethoxazole.

**Table 2:** Knowledge gaps and future directions.

Knowledge Gaps	Research Questions
**Pathogenicity of Mab** Is Mab virulence primarily driven by genetic factors, epigenetic regulation, or a combination of both?	Does CFTRm therapy induce molecular changes in Mab? Does CFTRm therapy affect virulence phenotypes, including antibiotic resistance and tolerance? Can molecular assays predict tolerance phenotypes to allow tailored antibiotic treatment?
**Host Immune Response in CF** **Is immune dysfunction in pwCF an intrinsic feature of the disease, or is it a consequence of the altered airway environment??**	Is Mab infection in CF epithelial cells affected by presence of wild-type versus CFTR-deficient macrophages? In what ways (if any) does CFTRm therapy affect the immune system?
**Pathogen interactions within the CF airway**	Does the presence of pathogens such as *P. aeruginosa* or *Staphylococcus aureus* affect Mab pathogenicity? Do they have a protective or promoting effect? Can certain antibiotics be utilized to alter the CF microbiome in order to inhibit Mab growth and prevent infection?
**Advanced and Novel Therapies**	What is the therapeutic potential of host-directed immune modulatory strategies in the management of Mab infection in pwCF? Is there potential for novel therapies to serve as alternatives to traditional antibiotic treatment?

Mab = *Mycobacterium abscessus*; CF = cystic fibrosis; pwCF = people with cystic fibrosis; CFTR = cystic fibrosis transmembrane regulator; CFTRm = CFTR modulators.

## Data Availability

No new data were created or analyzed in this study.
